# Seroprevalence and Distribution of *Toxoplasma gondii* Infections among Patients in the West Bank, Palestine (2017–2021)

**DOI:** 10.4269/ajtmh.23-0721cor

**Published:** 2025-03-05

In the original research paper, “Seroprevalence and Distribution of *Toxoplasma gondii* Infections among Patients in the West Bank, Palestine (2017–2021)” by Abu Seir et al., 2024, (https://doi.org/10.4269/ajtmh.23-0721), the authors missed to correct the data reported in the Abstract prior to publishing. The seroprevalence for IgG in the abstract was incorrectly recorded as 1.0%, whereas the correct value should be 21.0% (as rounded up from originally reported 20.95%). The paper has therefore been corrected. The results and conclusions are unchanged. A summary of the corrected data is shown below:

The overall prevalences of anti–*T. gondii* IgG and IgM antibodies were 21.0% [18.9–23.0%] and 1.0% [0.5–1.6%], respectively. (ABSTRACT, page 1000)

